# A novel mutation in *MIP* associated with congenital nuclear cataract in a Chinese family

**Published:** 2011-01-08

**Authors:** Kai Jie Wang, Sha Sha Li, Bo Yun, Wen Xian Ma, Tian Ge Jiang, Si Quan Zhu

**Affiliations:** Beijing Tongren Eye Center, Beijing Tongren Hospital, Capital Medical University, Beijing Ophthalmology & Visual Sciences Key Lab, Beijing, China

## Abstract

**Purpose:**

To identify the underlying genetic defect in a Chinese family affected with autosomal dominant congenital nuclear cataract.

**Methods:**

A four-generation Chinese family with inherited nuclear cataract phenotype was recruited. Detailed family history and clinical data were recorded. All reported nuclear cataract-related candidate genes were screened for causative mutations by direct DNA sequencing. Effects of amino acid changes on the structure and function of protein were predicted by bioinformatics analysis.

**Results:**

All affected individuals in this family showed nuclear cataracts. Sequencing of the candidate genes revealed a heterozygous c.559C>T change in the coding region of the major intrinsic protein (*MIP*), which caused a substitution of highly conserved arginine by cysteine at codon 187 (p.R187C). This mutation co-segregated with all affected individuals and was not observed in unaffected family members or 110 ethnically matched controls. Bioinformatics analysis showed that the mutation was predicted to affect the function and secondary structure of MIP protein.

**Conclusions:**

This study identified a novel disease-causing mutation p.R187C in *MIP* in a Chinese cataract family, expanding the mutation spectrum of *MIP* causing congenital cataract.

## Introduction

Congenital cataract is characterized by any opacities of the lens presented at birth or shortly thereafter. It is one of the most common disorders in the eye and a leading cause of blindness in children, with a prevalence of 1 to 6 cases/10,000 live births worldwide [1]. The cataract may be isolated, be associated with other developmental abnormalities of the eye, or part of an inherited multisystem disorder [2]. Approximately half of cataracts are genetically determined. Most congenital cataracts are usually inherited as autosomal dominant traits with almost complete penetrance and highly variable expressivity, although autosomal recessive and X-linked inheritance are also found [3].

Most progress has been made in identifying the genes causing autosomal dominant congenital cataract (ADCC) [4]. So far, more than 30 independent loci and at least 18 disease-causing genes have been identified to be responsible for isolated ADCC [5]. Among these genes, αA-crystallin (*CRYAA*), βA1-crystallin (*CRYBA1*), βB1-crystallin (*CRYBB1*), βB2-crystallin (*CRYBB2*), γC-crystallin (*CRYGC*), γD-crystallin (*CRYGD*), connexin 46 (*CX46*), connexin 50 (*CX50*), and major intrinsic protein (*MIP)* are highly expressed in the lens and have been shown to be associated with nuclear cataract [1,4], and thus represent excellent candidate genes for hereditary nuclear cataracts.

In the present study, due to limited family members participated in this study, we applied a functional candidate approach testing for the nine well known cataract-causing genes in a Chinese family. A novel missense mutation in *MIP* that co-segregated with the disease was identified to be responsible for nuclear cataract.

## Methods

### Clinical evaluation and DNA specimens

This study was conducted in accordance with the tenets of the Declaration of Helsinki and approved by the ethics committees for medical research at Capital Medical University, Beijing, China. A four-generation family with ADCC was recruited at Beijing Tongren Hospital (Capital Medical University, Beijing, China). Informed consent was obtained from all participants of the family, originating from the province of Heilongjiang in the Northeast of China. Affected status was determined by a medical history or ophthalmologic examinations, including visual acuity, slit lamp examination, ultrasonography, fundus examination and intraocular pressure measurement. The phenotypes were documented by slit lamp photography. A total of 110 unrelated control participants with no family history of congenital cataracts were also recruited. They were given complete ophthalmologic examinations as the participants of the cataract family and did not have eye diseases except mild myopia and senile cataracts. Peripheral venous blood was collected for genomic DNA extraction using QIAamp DNA kit (Qiagen, Valencia, CA) using standard protocols as previously described [6].

### Mutation analysis

Mutation screening was performed in the nine candidate genes, including *CRYAA* (GenBank NM_000394), *CRYBA1* (GenBank NM_005208), *CRYBB1* (GenBank NM_001887), *CRYBB2* (GenBank NM_000496), *CRYGC* (GenBank NM_020989), *CRYGD* (GenBank NM_006891), *CX46* (GenBank NM_021954), *CX50* (GenBank NM_005267) and *MIP* (GenBank NM_012064.3). All coding exons and splice sites of the candidate genes were amplified by polymerase chain reactions (PCR) using previously published primer sequences (Table 1)  [7-9]. The PCR products obtained from the proband and one unaffected member were sequenced on an ABI3730 Automated Sequencer (PE Biosystems, Foster City, CA). The sequencing results were analyzed using Chromas 2.33 and compared with the reference sequence in the NCBI database. Direct sequencing was also used to screen the mutation identified in *MIP* on the sample of all available family members and 110 ethnically matched controls to confirm the mutation.

**Table 1:** Primer sequences for PCR amplification.

**Gene**	**Forward primers (5′→3′)**	**Reverse primers (5′→3′)**	**Annealing temperature (°C)**	**Product size (bp)**
*CRYAA*-1	AGCAGCCTTCTTCATGAGC	CAAGACCAGAGTCCATCG	62	584
*CRYAA*-2	GGCAGGTGACCGAAGCATC	GAAGGCATGGTGCAGGTG	62	550
*CRYAA*-3	GCAGCTTCTCTGGCATGG	GGGAAGCAAAGGAAGACAGA	62	511
*CRYBA1*-1	GGCAGAGGGAGAGCAGAGTG	CACTAGGCAGGAGAACTGGG	60	550
*CRYBA1*-2	AGTGAGCAGCAGAGCCAGAA	GGTCAGTCACTGCCTTATGG	60	508
*CRYBA1*-3	AAGCACAGAGTCAGACTGAAGT	CCCCTGTCTGAAGGGACCTG	60	463
*CRYBA1*-4	GTACAGCTCTACTGGGATTG	ACTGATGATAAATAGCATGAACG	60	355
*CRYBA1*-5	GAATGATAGCCATAGCACTAG	TACCGATACGTATGAAATCTGA	60	597
*CRYBA1*-6	CATCTCATACCATTGTGTTGAG	CATCTCATACCATTGTGTTGAG	62	528
*CRYBB1*-1	CCCTGGCTGGGGTTGTTGA	TGCCTATCTGCCTGTCTGTTTCTC	58	620
*CRYBB1*-2	TAGCGGGGTAATGGAGGGTG	AGGATAAGAGTCTGGGGAGGTGG	58	664
*CRYBB1*-3	CCTGCACTGCTGGCTTTTATTTA	TCTCCAGAGCCCAGAACCATG	60	475
*CRYBB1*-4	CCAACTCCAAGGAAACAGGCATA	CCTCCCTACCCACCATCATCTC	60	491
*CRYBB1*-5	TAGACAGCAGTGGTCCCTGGAGA	AGCACTGGGAGACTGTGGAAGG	60	416
*CRYBB1*-6	CCTAGAAAAGGAAACCGAGGCC	AGCGAGGAAGTCACATCCCAGTA	60	551
*CRYBB2*-1	GTTTGGGGCCAGAGGGGAGTGGT	TGGGCTGGGGAGGGACTTTCAGT	62	349
*CRYBB2*-2	CCTTCAGCATCCTTTGGGTTCTCT	GCAGTTCTAAAAGCTTCATCAGTC	62	330
*CRYBB2*-3	GTAGCCAGGATTCTGCCATAGGAA	GTGCCCTCTGGAGCATTTCATAGT	62	360
*CRYBB2*-4	GGCCCCCTCACCCATACTCA	CTTCCCTCCTGCCTCAACCTAATC	62	230
*CRYBB2*-5	CTTACCCTTGGGAAGTGGCAATGG	TCAAAGACCCACAGCAGACAAGTT	62	600
*CRYGC*-1	TGCATAAAATCCCCTTACCG	CCTCCCTGTAACCCACATTG	62	514
*CRYGC*-2	TGGTTGGACAAATTCTGGAAG	CCCACCCCATTCACTTCTTA	60	430
*CRYGD*-1	CAGCAGCCCTCCTGCTAT	GGGTCCTGACTTGAGGATGT	60	550
*CRYGD*-2	GCTTTTCTTCTCTTTTTATTTCTGG	AAGAAAGACACAAGCAAATCAGT	62	308
*CX46*-1	CGGTGTTCATGAGCATTTTC	CTCTTCAGCTGCTCCTCCTC	60	450
*CX46*-2	GAGGAGGAGCAGCTGAAGAG	AGCGGTGTGCGCATAGTAG	60	450
*CX46*-3	TCGGGTTCCCACCCTACTAT	TATCTGCTGGTGGGAAGTGC	62	300
*CX50*-1	CCGCGTTAGCAAAAACAGAT	CCTCCATGCGGACGTAGT	62	420
*CX50*-2	GCAGATCATCTTCGTCTCCA	GGCCACAGACAACATGAACA	62	330
*CX50*-3	CCACGGAGAAAACCATCTTC	GAGCGTAGGAAGGCAGTGTC	62	350
*CX50*-4	TCGAGGAGAAGATCAGCACA	GGCTGCTGGCTTTGCTTAG	62	500
*MIP*-1	GTGAAGGGGTTAAGAGGC	GGAGTCAGGGCAATAGAG	62	561
*MIP*-2	CGGGGAAGTCTTGAGGAG	CACGCAGAAGGAAAGCAG	58	847
*MIP*-3	CCACTAAGGTGGCTGGAA	CTCATGCCCCAAAACTCA	60	561

### Bioinformatics analysis

The CLC Free Workbench 4.5.1 software (CLC bio, Aarhus, Denmark) was used to align the protein sequences from several different species. The isoelectric point (pI) and molecular weight (MW) of the wild type and mutant protein were analyzed by Compute pI/MW provided in the Expasy proteomics server. The possible functional impact of an amino acid change was predicted by Polymorphism Phenotyping (Polyphen) and Sorting Intolerant from Tolerant (SIFT). The secondary structure of mutant and wild-type amino acid sequences were analyzed by Antheprot 2000 V 6.0 software (IBCP, Lyon, France).

## Results

### Clinical findings

**Figure 1:**
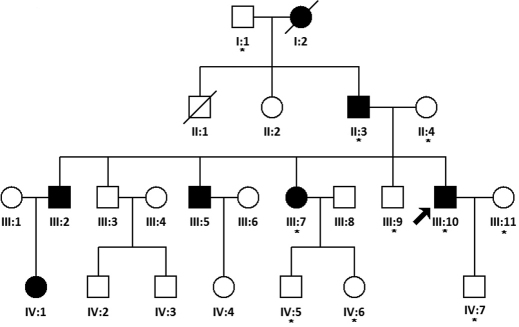
Pedigree of a Chinese cataract family. Pedigree of a four-generation family with autosomal dominant cataract. The black arrow indicates the proband. The asterisk indicates family members who attend this study.

**Figure 2:**
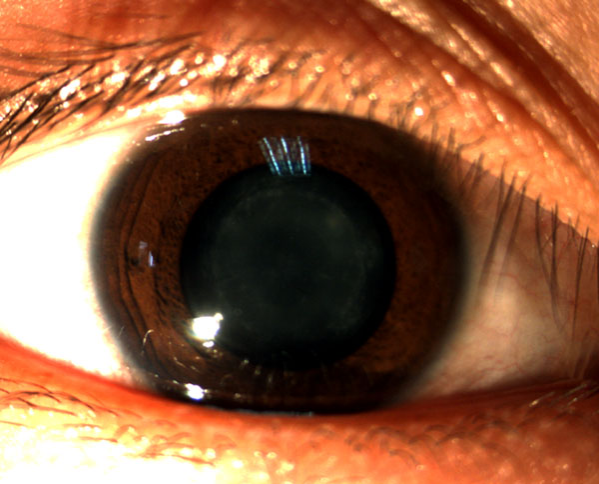
Slit lamp photographs of the proband. The photograph of the proband (III:10) shows nuclear opacities of the lens involving embryonic and fetal nucleus.

We identified a four-generation Chinese family with autosomal dominant nuclear cataract (Figure 1). In total 10 members (3 affected and 7 unaffected) participated in the study. The proband (III:10) was dignosed with bilateral nuclear cataract at the age of 18. The nuclear opacities were located in the embryonic and fetal nucleus (Figure 2). His best corrected visual acuity was 0.4 in both eyes. Individual II:3 was 61 years old and first diagnosed with bilateral nuclear cataract at the age of 21. The lens opacity was similar in terms of size and density in the two affected members and did not result in significant loss of visual acuity. According to the medical records, individual III:7 was first diagnosed with bilateral nuclear cataract and had cataract extraction performed at the age of 16 years. Other affected family members were diagnosed after the age of 10 years. There was no family history of other ocular or systemic abnormalities in this family.

### Mutation analysis

**Figure 3:**
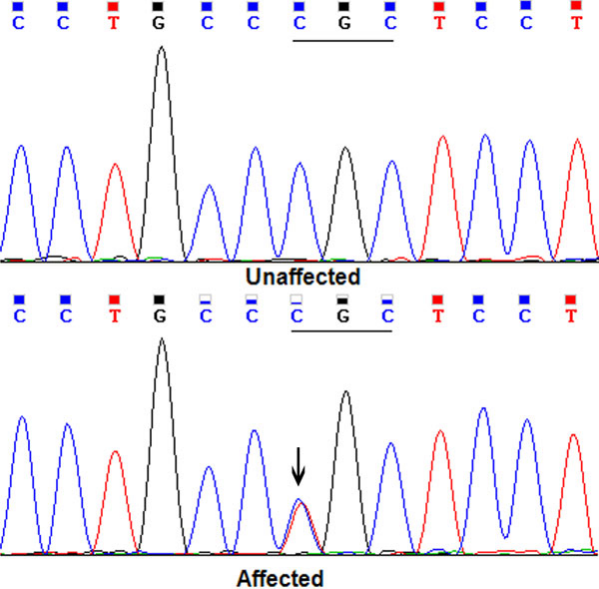
DNA sequence chromatograms of an unaffected member and an affected member in the family (Forward strand; individual III:9 and III:10, respectively). A single transition is observed at position 559(C>T) as a C/T double peak (indicated by an arrow).

Direct sequencing of the coding regions of the candidate genes in 3 affected individuals identified a single base alteration c.559C>T in exon 3 of *MIP* (Figure 3), which resulted in a substitution of arginine to cysteine at codon 187 (p.R187C). The substitution was not found in any of the unaffected family members or in the 110 unrelated controls from the same Northeastern Chinese population (data not shown). No other mutations were found except for a few non-pathogenic single nucleotide polymorphisms (SNPs).

### Bioinformatics analysis

**Figure 4:**
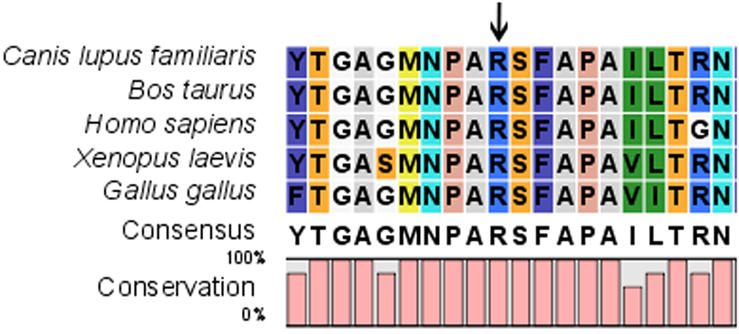
Multiple-sequence alignment in MIP from different species. A multiple alignment of partial amino acid sequences of MIP from different species is shown. The alignment data indicates that the Arg at position 187 is highly conserved in different species (indicated by an arrow).

**Figure 5:**
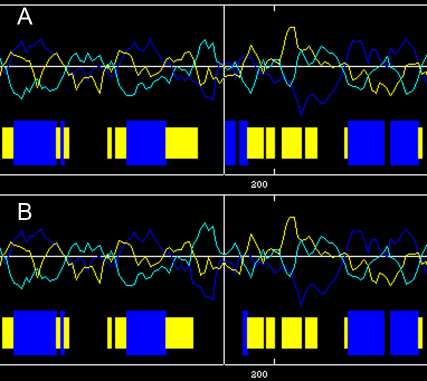
The predicted secondary structures of the mutant and the wild type amino acid sequences. The predicted secondary structures of the wild-type amino acid sequence (**A**) and the mutant amino acid sequence (**B**) is shown. The target sequences are labeled by the solid line, which indicate that the original α-helix is replaced by a coil in the mutant type. Blue: helix; Yellow: sheet; Black: coil.

The Arg at position 187 of human MIP was located within a phylogenetically conserved region by multiple-sequence alignment (Figure 4). The p.R187C MIP was predicted to be “probably damaging” by Polyphen with a score of 3.145, and “affect protein function” by SIFT with a score of 0.00. The theoretical pI of p.R187C MIP was reduced to 7.78 compared to wild type MIP pI of 8.62. The MW of the mutant was slightly reduced to 28068 Da from the MW of wild type MIP of 28121 Da. The secondary structure prediction showed that the mutation p.R187C led to the replacement of an original α-helix by a coil, a significant difference in coding position 187 of the secondary structure of MIP protein (Figure 5).

## Discussion

In the present study, we identify a novel p.R187C substitution in *MIP* associated with autosomal dominant nuclear cataract. The substitution segregates with the disease phenotype and is not observed in the unaffected family members or in the 110 ethnically matched controls. It is also predicted to be deleterious by both programs Polyphen and SIFT with consistent results. We, therefore, consider this variation as a causative mutation.

MIP, a membrane intrinsic protein expressed as a 263 amino acids (also known as AQP0), is inserted in the plasma membrane, contributing over 50% of the total membrane protein [10]. It is a member of the aquaporins, a ubiquitous family of membrane water transport protein that confer rapid movements of water across cell membranes [11]. Aquaporins share a unique structure with six transmembrane bilayer-spanning domains (H1-H6), three extracellular loops (A, C, and E), two intracellular loops (B and D), and the NH_2_- and COOH-terminal intracellular domains [12]. A highly conserved asparagine–proline–alanine (NPA) motif is present in the loops B and E; the NPA motif folds back into the membrane and forms two hemipores (HB and HE), in accordance with the predicted “hourglass model” [13,14] that is later conﬁrmed by crystallography studies [15,16]. Besides functioning as a water channel, MIP has been found to play a structural and adhesive molecule role, being required for maintaining the lens transparency [17,18]. Mutations in MIP in human and mice and knockout mouse model have been shown to induce bilateral cataract [10,12,19], which further highlight the important role of this protein for normal lens growth.

The mutation identified here, p.R187C *MIP*, occurs in highly conserved amino acid located within the hemipore HE and lies close to the NPA motif. To our knowledge, this is the first mutation in the HE domain of MIP found to be disease-causing for ADCC. Arginine usually lies in the active or binding sites of the protein because of its multiple amino groups at the end of the side chain [20]. Thus, arginine may play a vital role on the conformation and function of proteins. Replacement of positively charged residue arginine (R187) with cysteine, a small but uncharged residue, eliminates a fixed charge lining the aqueous pathway. A reduction of theoretical isoelectric point from 8.62 to 7.78 is also noted as predicted by Compute pI, although its effect on the protein structure needs to be further investigated. This may alter the protein conformation and affect the formation of water pore channel and therefore water permeability. Moreover, the protein structure analysis by Antheprot 2000 V 6.0 clearly shows a significant change of the secondary structure around the Arg- Cys substitution site in MIP, which may subsequently prevent its oligomerization. As exhibited by biochemical and structural analyses, aquaporins are functional only in the tetrameric form in the membrane [21]. Hence, the predicted change of protein structure due to the p.R187C might leads to fail of forming the water channel or disturbance of water transport through the channel. Another possibility is that the protein may be misfolded and trapped in the endoplasmic reticulum, as observed for several mutant membrane proteins [22,23].

Mutations in the human *MIP* gene causing congenital cataract have been identified in eight families, as listed in Table 2 [9,20,24-27]. All the *MIP* mutations including p.R187C identified in this study present bilateral cataracts as the autosomal dominant phenotype, indicating the important structural role of MIP in the lens. The cataracts caused by *MIP* mutation are usually located in the nuclear region of the lens. The phenotype described in this study also shows marked nuclear cataract, indicating a good phenotype-genotype pattern in ADCC. In these mutations, three of them have been functionally characterized in vitro, providing insights into the molecular mechanism responsible for the dominant effect of the mutations. p.E134G and p.T138R mutations result in loss of water permeability due to the failure in trafﬁcking of the proteins to the plasma membrane. In addition, when the p.E134G or p.T138R mutant is co-expressed with wild-type MIP protein, the mutant protein reaches the plasma membrane but causes instability of the tetramers and loss of function in the wild-type MIP [28], consistent with a dominant negative mechanism for the autosomal dominant inheritance of the cataracts. The 638G deletion in *MIP* identified in an American family causes a frameshift leading to a truncated protein, and functional study shows that the mutant protein retains in the endoplasmic reticulum and induces cytotoxicity due to the accumulation of the mutant protein [29]. Therefore, all these results suggest the key role of MIP in physiologic functioning of the lens.

**Table 2:** Summary of identified mutations in *MIP* responsible for congenital cataract.

**Mutation**	**Amino acid change**	**Location**	**Cataract type**	**Origin of family**	**Reference**
c.97C>T	p.R33C	Loop A	Total cataract	Chinese	[9]
c.319G>A	p.V107I	Loop C	Nuclear	Chinese	[24]
c.401A>G	p.E134G	H4	Lamellar and sutural	English	[25]
c.413C>G	p.T138R	H4	Polymorphic	English	[25]
c.559C>T	p.R187C	HE	Nuclear	Chinese	Present study
IVS-1G>A		H6	Nuclear “snail-like”	Chinese	[26]
C.638delG	p.D213fs	H6	Polymorphic	American	[27]
c.702G>A	p.R233K	COOH-terminus	Polymorphic	Chinese	[20]

Shown are *MIP* mutations that have been identified in this and other studies.

In conclusion, we described a novel missense mutation (p.R187C) in *MIP* that causes ADCC in a Chinese family. The predicted change of the protein structure may affect the function of water channel in the lens. Further investigations are needed to provide further insights into the molecular mechanism of this mutation.
